# Electronic Voltage and Current Transformers Testing Device

**DOI:** 10.3390/s120101042

**Published:** 2012-01-18

**Authors:** Feng Pan, Ruimin Chen, Yong Xiao, Weiming Sun

**Affiliations:** Electric Power Research Institute of Guangdong Power Grid Corporation, No. 8 Shuijungang Dongfengdong Road, 510080 Guangzhou, China; E-Mails: RMCH@263.NET(R.C.); xiaoyong_gddky@163.com (Y.X.); swm2804@139.com (W.S.)

**Keywords:** EVTs, ECTs, testing device, ratio error, phase error

## Abstract

A method for testing electronic instrument transformers is described, including electronic voltage and current transformers (EVTs, ECTs) with both analog and digital outputs. A testing device prototype is developed. It is based on digital signal processing of the signals that are measured at the secondary outputs of the tested transformer and the reference transformer when the same excitation signal is fed to their primaries. The test that estimates the performance of the prototype has been carried out at the National Centre for High Voltage Measurement and the prototype is approved for testing transformers with precision class up to 0.2 at the industrial frequency (50 Hz or 60 Hz). The device is suitable for on-site testing due to its high accuracy, simple structure and low-cost hardware.

## 1. Introduction

Nowadays, there are a variety of types of instrument transformers based on different measurement principles in electric power systems. They can be based on air core coils [1], shunts [2], capacitors [3], resistors [4], optical principles (e.g., Faraday’s and Pockel’s effects [5]) and so on. Their outputs have different forms, from voltage and current analog signals to digital signals in various formats. The rated secondary outputs of conventional transformers are voltage with a value of 100 or $100/\sqrt{3}$ V and current with 1 or 5 A. The EVTs/ECTs have analog voltage outputs or/and digital outputs. For EVTs/ECTs with analog outputs, the values of rated secondary voltage are in the range from 22.5 mV to 6.5 V. The digital output is a data stream coming from the merging unit (MU) which supplies the electrical instruments and electrical protective devices with digitally coded time-coherent sets of voltage/current data [6–9]. Such diversity makes it difficult to test all kinds of instrument transformers using a single device.

The purpose of testing the instrument transformers is to obtain the ratio error and phase error of the transformer under test under certain conditions. In general, the errors are determined by comparing the output of the tested transformer and the output of the reference transformer when the same signal is applied to their primary. For conventional transformers, the operation is usually carried out by the transformer comparator (TC) [10]. The TC has complex electromagnetic section to assure good accuracy [11]. The high cost, large size and heavy weight of the TC prohibit its on-site use.

With some modifications, the TC can also be used for testing EVTs/ECTs with analog outputs. Another method is to transform the analog outputs of the reference transformer and the tested transformer into digital signals, and to perform the comparison in the digital domain [12,13].

For digital outputs of EVTs/ECTs, one of the possible approaches is to convert the digital output into an analog one and to take measures by the TC method, but the time delay introduced by the repeated conversions will cause a phase displacement that is outside the handling range of the TC. In addition, this method adds to the uncertainty and it is not directly possible to be sure that the data stream is in accordance with the standards [14]. It is also possible to transform the analog output of the standard transformer into digital signal and compare with the digital output of the EVTs/ECTs [15].

Each of these testing devices can only test one type of transformer or a few. Some devices are not suitable for on-site testing due to the high-cost hardware, complex structure and huge size of the instruments.

Owing to the above considerations, a testing device is presented in this paper. It is able to fulfill the testing requirements of electronic instrument transformers with analog and digital outputs. Since the testing device has a simple structure and uses low-cost hardware, it can be easily built and is suitable for on-site testing.

## 2. Testing Principal for Electronic Voltage and Current Transformers

### 2.1. Testing Principal

**Figure 1.**
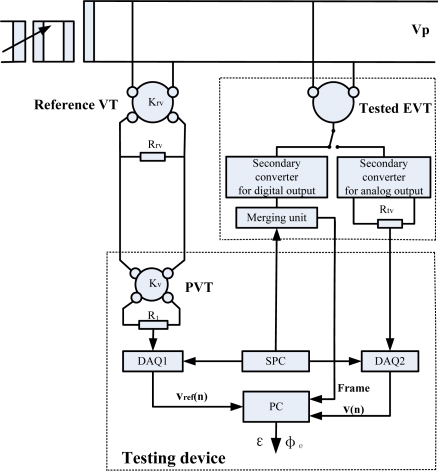
Block diagram of testing EVT.

**Figure 2.**
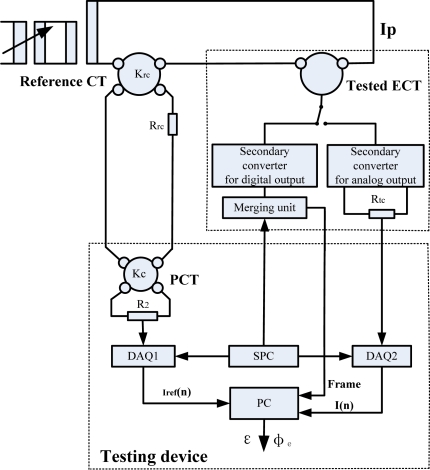
Block diagram of testing ECT.

The block diagrams of testing EVTs and ECTs are shown in Figures 1 and 2, respectively. The testing device consists of a precision voltage transformer (PVT), precision current transformer (PCT), data acquisition boards (DAQ1 and DAQ2), synchronous sampling pulse circuit (SPC) and computer (PC).

The primaries of the transformer under test and the reference transformer are fed to the same signal (a sinusoidal voltage or current), whereas each secondary is connected to the required burden. The PVT is used to transform the secondary voltage of the reference VT to a low-voltage, which is suitable for the input range of the DAQ1. The PCT is used to transform the secondary current of the reference CT to a low-current, which causes a voltage drop across the resistor R_2_ and the voltage signal is sampled by the DAQ1.

For the analog output of EVT/ECT testing, the secondary voltage output is directly sampled by DAQ2. Compared to the analog output of EVT/ECT, the digital output is not a voltage signal but a digital data stream. The data stream is captured by the PC and the Winpcap application translates it in the LabVIEW programming environment.

In the testing process, a time base is important to ensure time-coherent data sets from the tested transformer and the reference transformer. The SPC generates a pulse-per-second (PPS) signal and it can be used for this purpose. Since the transformer under test and the reference transformer are supplied with the same clock pulses, their samples can be compared directly. The error calculation can be made according to the mathematical method described in Section 2.6. The calculation procedures are also implemented in the Labview programming environment.

### 2.2. The Performance of Reference VT and CT

It is necessary to choose a reference VT and a reference CT with the precision class that is properly lower than the transformer under test. The errors of the reference VT and CT were determined by the TC method. The measurement uncertainty is less than 0.01% for ratio error and 0.3 min for phase error.

### 2.3. The Performance of PVT and PCT

The calibration of the PVT and PCT, which accommodates the input range requirements of the DAQ, was also done using the TC. The uncertainty of the PVT is found to be less than 0.005% for ratio error and 0.17 min for phase error. The uncertainty of the PCT is less than 0.01% for ratio error and 0.3 min for phase error.

### 2.4. The Performance of R_1_, R_2_

The R_1_ is used as the rated secondary burden of PVT. The R_2_ is used as the rated secondary burden of PCT and as the converter from current to voltage which supplies the DAQ1/DAQ2 with proper voltage value. These resistors were tested by the precision impedance analyzer (WAYNE KERR 6500B). The relative errors of the resistance are less than 0.01% and the inductive impedances are less than 0.1 μH at the industrial frequency.

### 2.5. The Performance of DAQ1 and DAQ2

The testing device requires not only high resolution but also low uncertainty. To assure low uncertainty, two commercially data acquisition boards (DAQs) are used. They achieve low-uncertainty performance and constrain the sampling to 5 kS/s for 18-bit resolution. The DAQs have a reaction time which is equivalent between the moment the trigger clock is given and the moment the first sample is taken. The delay time has an uncertainty of 500 ns, thus the phase uncertainty is 0.54 min using 50 Hz as the frequency. The quantization error and nonlinearity error of the AD converters in the data acquisition boards can be neglected since their effects are usually negligible [16].

### 2.6. Mathematical Method for the Error Calculation

A Discrete Fourier Transform (DFT) algorithm is employed to extract the amplitude and phase at the frequency of the excitation signal. The differences of the values between the two signals from the reference transformer and the tested transformer are the ratio error and phase error, namely, the differences between the two values that are given by the DFT algorithm.

Take the EVT testing for example. Both *v_ref_*(*n*) and *v*(*n*) are periodical signals. The Fourier transform of these signals after digitization are given by the formula:

(1) $$V_{\mathrm{ref}}=\sum_{n=0}^{\mathrm{kT}/T_{S}-1}\;v_{\mathrm{ref}}\;\left(n\right).e^{-j.2\pi f.n}$$

(2) $$V_{t}=\sum_{n=0}^{\mathrm{kT}/T_{S}-1}\;v\left(n\right).e^{-j.2\pi f.n}$$

where *v_ref_*(*n*) is the digital output of the DAQ1, *v*(*n*) is the digital output of the DAQ2, *n* is the data set counter, *k* is the summation periods, *T_S_* is the distance in time between two samples of the primary voltage.

Ratio error is expressed by:

(3) $$\varepsilon \;\left(\%\right)=100\cdot \frac{k_{\mathrm{tv}}\left|V_{t}\right|-k_{\mathrm{rv}}\;k_{v}\left|V_{\mathrm{ref}}\right|}{k_{\mathrm{rv}}\;k_{v}\left|V_{\mathrm{ref}}\right|}$$

where *k_rv_* is the transformation ratio of the reference transformer, *k_v_* is the transformation ratio of PVT, *k_tv_* is the transformation ratio of the tested transformer.

Phase error is expressed by:

(4) $$\Phi_{e}=\Phi_{t}-\Phi_{\mathrm{ref}}$$

where Φ*_t_* is the phase of *V_t_*, Φ*_ref_* is the phase of *V_ref_*.

The errors that are introduced by the algorithm have to be considered. Since the measurement of the amplitude and the phase are based on the DFT algorithm, leakage effects happen when the sampling rate is not an exact multiple of the frequency of the voltage or current that is applied to the primary of the reference and tested transformers. The windows technique and phase difference correction method can greatly reduce the effects [17,18].

## 3. Performance Evaluation of the Testing Device

A prototype of testing device is developed according to the proposed method. The transformation ratio of PVT is $100/\sqrt{3}$ V : $4/\sqrt{3}$ V at the rated burden R1 (8 kΩ). The transformation ratio of PCT is 5 A : 0.05 A at the rated burden R2 (80 Ω). For a rated 50-Hz signal measurement, a sampling frequency of 4,000 Hz and 800 samples are adopted in the error calculation procedures.

To evaluate the performance of the prototype, some tests have been performed at the National Center for High Voltage Measurement. In particular, these tests have been mainly focused on the section of prototype including PVT, PCT, DAQ1, DAQ2 and the algorithm.

**Figure 3.**
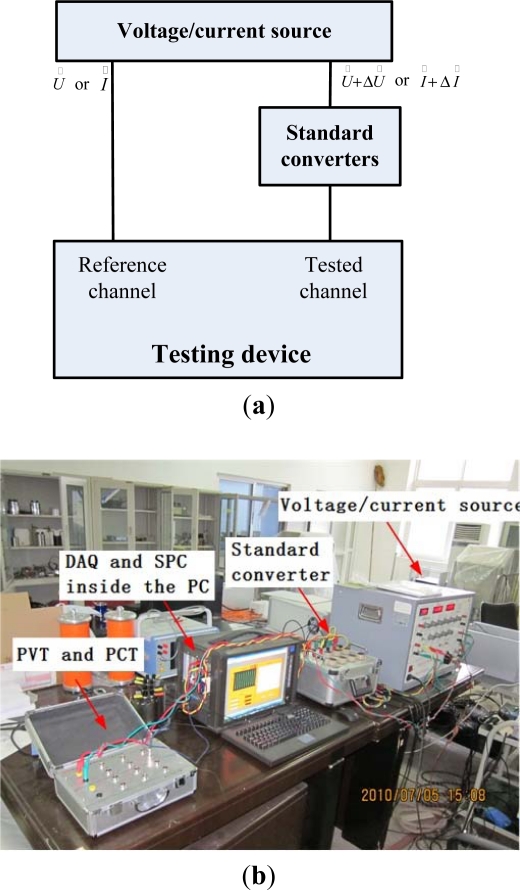
(**a**) Test setup for verification of the testing device. (**b**) Photo of the experimental setup.

The test setup for verification of the testing device is shown in Figure 3. A high precision excitation source generates two sinusoidal voltage or two sinusoidal current signals, which have the same frequency but different amplitude and phase values, namely, $\Delta \overset{g}{U}/\overset{g}{U}$ and $\Delta \overset{g}{I}/\overset{g}{I}$ are adjustable.

The reference channel includes the PVT, PCT and DAQ1. The tested channel consists of the DAQ2 and network card. For evaluating the performance of the prototype, using both channels of the source and varying the relative amplitude and phase, it is possible to obtain the difference between the calculated value of the prototype under evaluation and the set value of the source.

In order to evaluate the performance of EVTs/ECTs testing device, the $\overset{g}{U}$ and $\overset{g}{U}+\Delta \overset{g}{U}$ (or $\overset{g}{I}$, $\Delta \overset{g}{I}+\overset{g}{I}$) can feed into the reference channel and the tested channel, respectively. For measuring and metering purpose introduced in the IEC standard (IEC 60044-7, IEC 60044-8), the ratio error and phase error at the voltage between 80% and 120% of the rated voltage and at the current between 5 % and 120% of the rated current, should be taken into consideration. As a result, in the evaluation of the testing device, the rated value of testing voltage is $100/\sqrt{3}$ V (*U_rated_*) which is equal to the rated output voltage of the reference voltage transformer and the tested voltage values are 80% *U_rated_*, 100% *U_rated_*, 120% *U_rated_*, The rated value of testing current is 5 A (*I_rated_*) which is equal to the rated output current of the reference current transformer and the tested current values are 5% *I_rated_*, 20% *I_rated_*, 100% *I_rated_*, 120% *I_rated_*.

The standard converters are necessary to convert the high analog signals into low ones or into digital data streams, which comply with the input range of the tested channel or the Ethernet frame format. The standard converters consist of an inductive voltage divider, a shunt, a data multimeter (DMM) and an Ethernet interface. Their uncertainties of the main components are shown in Table 1.

Take the EVT testing device for example. The ratio error of the testing device under evaluation is expressed by:

(5) $$\begin{array}{l} \varepsilon =\frac{\left(U+\Delta U\right)\left(1+b\right)\left(1+c\right)-U\left(1+a\right)}{U\left(1+a\right)}-\frac{\Delta U}{U}\;\left(1+d\right)\\ =\frac{b+c+\mathrm{bc}-a}{1+a}+\frac{\Delta U}{U}\cdot \frac{b+c-a-d+\mathrm{bc}-\mathrm{ad}}{1+a} \end{array}$$

where *a*, *b*, *c*, *d* are the relative errors of the reference channel, tested channel, standard converter and Δ*U*/*U*, respectively.

For a << 1, b << 1, c << 1 and d << 1, then:

(6) $$\varepsilon \approx b-a+c+\frac{\Delta U}{U}\cdot \left(b+c-a-d\right)$$

For Δ*U*/*U* << 1, we have:

(7) ε≈b−a+c

The phase error of the testing device is expressed by:

(8) $$\begin{array}{l} \varphi_{\mathrm{cal}}=\left(\varphi_{U}+\Delta \varphi_{U}+\Delta \varphi_{r}\right)-\left[\varphi_{U+\Delta U}+\Delta \varphi_{U+\Delta U}+\Delta \varphi_{\mathrm{sc}}+\Delta \varphi_{t}\right]-\left(\varphi_{U}-\varphi_{U+\Delta U}\right)\\ =\Delta \varphi_{r}-\Delta \varphi_{t}-\Delta \varphi_{\mathrm{sc}}+\left(\Delta \varphi_{U}-\Delta \varphi_{U+\Delta U}\right) \end{array}$$

where Δ*φ_U_*, Δ*φ*_*U*+Δ*U*_, Δ*φ_r_*, Δ*φ_SC_*, Δ*φ_t_*, are the phase errors of the $\overset{g}{U}$, $\overset{g}{U}+\Delta \overset{g}{U}$, reference channel, standard converter and tested channel, respectively.

For (Δ*φ_U_* − Δ*φ*_*U*+Δ*U*_) ≈ 0, then:

(9) $$\varphi_{\mathrm{cal}}=\Delta \varphi_{r}-\Delta \varphi_{t}-\Delta \varphi_{\mathrm{sc}}$$

Equations (8) and (9) permit us to evaluate the performance of the testing device using the method with less accurate source but more accurate standard converters.

**Figure 4.**
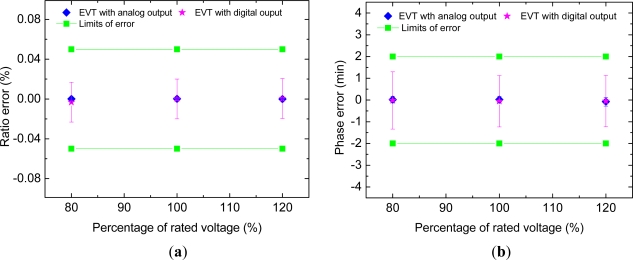
(**a**) Percentage ratio error of testing EVT. (**b**) Phase error of testing EVT.

**Figure 5.**
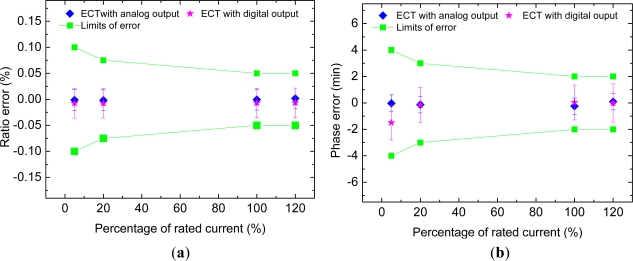
(**a**) Percentage ratio error of testing ECT. (**b**) Phase error of testing ECT.

Performance testes have been carried out at different values of $\Delta \overset{g}{U}/\overset{g}{U}$ and $\Delta \overset{g}{I}/\overset{g}{I}$. For the sake of simplicity, only the results that are obtained under the condition of $\Delta \overset{g}{U}/\overset{g}{U}=0$ and $\Delta \overset{g}{I}/\overset{g}{I}=0$ have been presented. The percentage ratio error and phase error of the testing device are shown in the Figures 4 and 5. In these figures, the error bars indicate the overall measurement uncertainty (with a covering factor of 2) due to the standard converters and the measurement repeatability of the system (type A uncertainty evaluation). As indicated clearly, the errors of the testing device under evaluation are less than the limits of ratio error and phase error for 0.05 class and hence sufficient for testing transformers with precision classes up to 0.2.

## 4. Conclusions

In this paper, a testing device for electronic voltage and current transformers has been introduced and the metrological characterization of the device has been discussed. The device is based on the digital signal processing technology, which can test both analog and digital outputs. The performance tests show that the device is suitable for the testing transformers with precision classes up to 0.2 at the frequency of 50 Hz or 60 Hz.

## Figures and Tables

**Table 1.** Uncertainties of the standard converters.

**Device**	**Magnitude (%)**	**Phase (min)**
**Inductive voltage divider**	0.0002	0.069
**Shunt**	0.01	0.3
**DMM**	0.01	0.583
